# Supplementary material for: Identification of photoperiod-induced specific miRNAs in the adrenal glands of Sunite sheep (Ovis aries)
Source: Front Vet Sci. 2022 Jul 22;9:888207. doi: 10.3389/fvets.2022.888207 (PMC9354845; doi:10.3389/fvets.2022.888207)
Supplement: Supplementary file 1 [file Data_Sheet_1.ZIP › Supplementary file/miRDeep2 file.zip!/miRDeep2 file/Novel_Structure/LP42A1/Novel_12.pdf]

Provisional ID : NC_019464.2_14397
Score total : 88.5
Score for star read(s) : 3.9
Score for read counts : 81.2
Score for mfe : 1.8
Score for randfold : 1.6
Score for cons. seed :
Total read count : 171
Mature read count : 156
Loop read count : 0
Star read count : 15

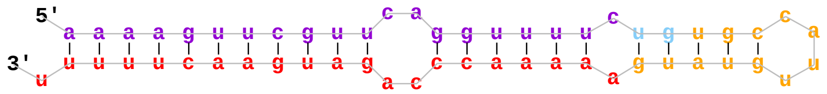

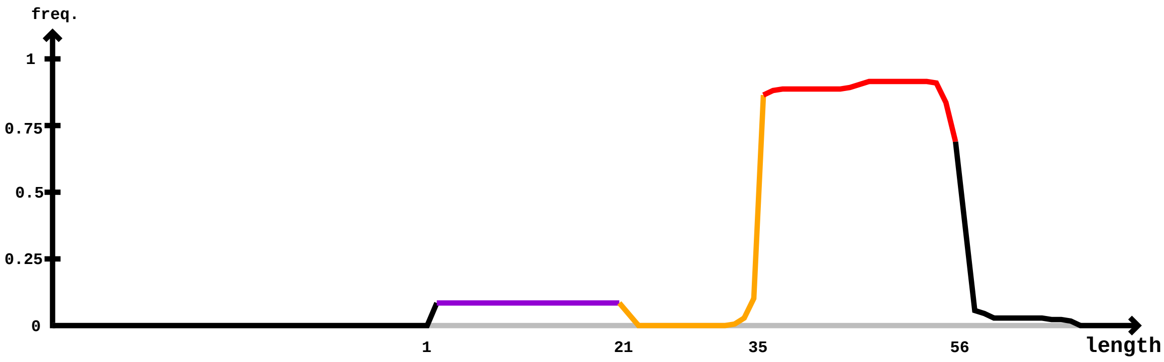


```
                                          Star                             Mature
5'- uguaccaaugaguauccuuguaugcacuggguugaccaaaaaguucguucagguuuucugugccauuguaugaaaaacccagaugaacuuuuuggccaacccaauacauaa   -3'   obs
    uguaccaaugaguauccuuguaugcacuggguugaccaaaaaguucguucagguuuucugugccauuguaugaaaaacccagaugaacuuuuuggccaacccaauacauaa         exp
    .((((......))))...(((((....(((((((.((((((((((((((..((((((.(((((....))))).))))))..)))))))))))))).)))))))))))...   reads   mm   sample
    ......................................aaaaguucguucGgguuuuc.....................................................   15      1    seq
    .....................................................................augaaaaacccaAaugaacuu.....................   1       1    seq
    ......................................................................ugaaaaacccagaugaacuuu....................   1       0    seq
    ......................................................................Agaaaaacccagaugaacuuuu...................   3       1    seq
    .......................................................................gaaaaacccagaugaacuuuu...................   13      0    seq
    ........................................................................aaaaacccagaugaacuuu....................   10      0    seq
    ........................................................................aaaaacccagaugaacuuC....................   2       1    seq
    ........................................................................aaaaacccagaugaacuuuu...................   9       0    seq
    ........................................................................aaaaacccagaugaacuuuC...................   1       1    seq
    ........................................................................aaaaacccagaugaacuuuuu..................   61      0    seq
    ........................................................................aaaaacUcagaugaacuuuuug.................   30      1    seq
    ........................................................................aaaaacccaAaugaacuuuuug.................   16      1    seq
    ........................................................................aaaaacccagaugaacuuuuug.................   4       0    seq
    ........................................................................aaaaacUcagaugaacuuuuugg................   2       1    seq
    .........................................................................aaaacccagaugaacuuuuu..................   1       0    seq
    .........................................................................aaaacUcagaugaacuuuuuggc...............   2       1    seq
    ..........................................................................aaacUcagaugaacuuuuuggc...............   1       1    seq
    .................................................................................Uaugaacuuuuuggccaacccaa.......   1       1    seq
    ..................................................................................augaacuuuuuggccaaccc.........   1       0    seq
    ..................................................................................augaacuuuuuggccaacccaaG......   1       1    seq
    ...................................................................................ugaacuuuuuggccaacccaau......   2       0    seq
```
